# Difference in bypass for inpatient care and its determinants between rural and urban residents in China

**DOI:** 10.1186/s12939-022-01734-0

**Published:** 2022-09-13

**Authors:** Shan Lu, Yunxiao Li, Hongxia Gao, Yan Zhang

**Affiliations:** 1 School of Medicine and Health Management, Tongji Medical College, Huazhong University of Science and Technology, Wuhan, 430030 China; 2 Research Centre for Rural Health Service, Key Research Institute of Humanities & Social Sciences of Hubei Provincial Department of Education, Wuhan, 430030 China

**Keywords:** Bypass behavior, Inpatient care, Urban-rural differences, China

## Abstract

**Background:**

Bypass for inpatient care is an event of excessive demand. Though primary care facilities provide inpatient care in China, local residents may choose more distant higher-level hospital for inpatient care services. Given the differences in accessibility of hospitals and socioeconomic development between urban and rural areas, this study aims to estimate the rate of bypass for inpatient care and explore the factors predictive of bypass among rural and urban residents in China.

**Methods:**

The rates of bypass for inpatient care were estimated using data from 1352 hospitalized patients, obtained from the 2018 Sixth National Health Service Survey of Hubei, China. Bypass for inpatient care was identified if the patient was hospitalized in a hospital for a certain disease that should be treated at primary care facilities in accordance with government requirement. Anderson’s Behavioral Model of Health Services Use was used as a theoretical framework for determining the factors of bypass. Logistic regression was used to identify the relationship between bypass for inpatient care and predisposing, enabling, and need characteristics for urban and rural residents.

**Results:**

The rate of bypass for inpatient care was 73.8%. This rate for inpatient care (91.3%) in cities is higher than that in rural areas (56.2%). Age were associated with bypass for both rural (OR, 0.982; 95% CI, 0.969–0.995) and urban (OR, 0.947; 95% CI, 0.919–0.976) patients. The patients whose closest healthcare facility was hospitals were more likely to have bypass behavior in rural (OR, 26.091; 95% CI, 7.867–86.537) and urban (OR, 8.323; 95% CI, 2.936–23.591) areas than those living closest to township/community health centers. Signing a family doctor was not helpful for retaining patients at primary care facility. Among rural patients, those with circulatory (OR, 2.378; 95% CI, 1.328–4.258), digestive (OR, 2.317; 95% CI, 1.280–4.192), or skin and bone (OR, 1.758; 95% CI, 1.088–2.840) system diseases were more likely to show bypass behavior than those with respiratory diseases.

**Conclusions:**

Bypass for inpatient care is sizable, and urban residents have a higher bypass rate for inpatient care than rural residents in China. More actionable measures in strengthening and leading patients to primary care are needed. Gradual establishment of a referral system is recommended. Inpatient care for circulatory, digestive, or skin and bone system diseases may be prioritized to be improved at primary care facilities in rural China.

**Supplementary Information:**

The online version contains supplementary material available at 10.1186/s12939-022-01734-0.

## Background

In recent years, excessive demand on inpatient care has been continuously concerned by policy makers and health insurers [1]. This excessive demand on inpatient care is due to patients receiving unnecessary inpatient care or services that are beyond their capacity to pay [2]. It could occur on three occasions: inappropriate admission, where patients receive unnecessary inpatient care rather than outpatient one or services that are beyond their capacity to pay; inappropriate inpatient services, in which patients receive inappropriate services during necessary hospitalization; and bypass for inpatient care, in which patients unnecessarily receive inpatient care from a higher-level hospital located farther away than the health care facility closest to their residence [3]. This research mainly focuses on the last occasion. The concept of bypass for inpatient care emphasizes that the inpatient care received by patients is unnecessary. The bypass behavior for diseases or illnesses that could be treated at local healthcare facilities brings many disadvantages, as it undermines functions of local healthcare facilities, crowds out resources for patients in need of higher-level hospital care, increases economic burden of patients, and decreases the effectiveness of a healthcare system in the long run [4].

In China, healthcare facilities that provide clinical care could be roughly classified into two groups: primary care facilities and hospitals. Primary care facilities predominantly consist of village clinics and township health centers in rural areas and community health stations and community health centers in urban areas. Except for hospitals, primary care facilities, including township health centers and community health centers, also provides inpatient care to residents in each town or community. Standardized primary care inpatient services constitute services from department of internal medicine, pediatrics, surgery and obstetrics and gynecology, and the inpatient services are mainly provided by doctors, nurses, pharmacists, clinical laboratory technicians and imaging technicians who work at primary care facilities. Primary care inpatient services are funded by a combination of health insurance and out-of-pocket payments. Without a gate-keeping system, residents are free to choose their first-contact healthcare facility; they generally have little trust in primary care and usually bypass primary care facilities to seek healthcare in hospitals. The growth rate of outpatient visits and admissions at hospitals were much higher than that at township/community health centers [5], leading to “kan bing nan, kan bing gui” (“medical treatment is difficult to access and expensive”) for decades [6].

In response to the unbalanced distribution of medical resources and inappropriate patient flow, China issued policy documents on the Hierarchical Medical System in 2015 and family doctor signing service in 2016 [7, 8]. The Hierarchical Medical System indicates that different levels of healthcare facilities have a clear division of responsibilities for undertaking different health services. Family doctor signing service was introduced to promote the establishment of the Hierarchical Medical System through building a stable service relationship between primary care physicians (or teams) and contracted residents. Unlike the mandatory gatekeeping system by legislation in many countries [9], patients were voluntarily encouraged to sign a contract with primary care physicians (or team), visit primary care first, and utilize hospital care via referral from primary care physicians through economic incentives in China. This study focuses particularly on bypassing primary care for inpatient care at hospitals.

Studies from several countries have focused on bypass behavior, in which a patient travel past the local primary care facility to seek services further away [10–12]. The rates of bypassing primary care ranged from 13.7% in Japan [6] to 67% in India [8], implying that bypass behavior is context dependent; thus, country-specific analysis is indispensable [4]. Various factors are associated with bypass behavior, and they could be categorized into two groups: patient factors, such as income, satisfaction, and disease severity [13, 14]; and supply-side factors, including geographic accessibility, quality of care, and cost of treatment [10, 15]. Evidence regarding bypass for inpatient care from China is limited. Few studies related to bypass have only investigated the bypass rate for outpatient care [16], the distribution of health spending between primary and higher-level care [17, 18], and the factors associated with the health-seeking behavior of patients through qualitative analysis [19, 20]. To date, the rate of bypass for inpatient care and its determinants are largely unknown, and little is known with regard to the difference in bypass for inpatient care between rural and urban areas in China, especially after the implementation of the Hierarchical Medical System and family doctor signing service. Given the differences in accessibility of hospitals and economic development, differences possibly exist in the bypass for inpatient care between urban and rural areas. Therefore, this study aimed to estimate the rate of bypass for inpatient care and explore the factors predictive of bypass in rural and urban areas, respectively. Rural-urban comparison may be conducive to understanding the differences in health-seeking behaviors and improving equity in health care between rural and urban residents. Bypass for inpatient care is a type of health services utilization behavior. Anderson’s Behavioral Model, which provides a theoretical framework for explaining health services utilization behavior and was validated on rural and urban population in China in many previous studies [21–25], was used to identifying potential factors of bypass behavior.

## Materials and methods

### Study design and sample

Data based on cross-sectional questionnaire surveys among urban and rural residents were derived from the sixth National Health Service Survey (NHSS) of Hubei Province in 2018. The NHSS is a 5-yearly survey administered by the Center for Health Statistics and Information of the National Health Commission. Multi-stage stratified cluster random sampling was used to select samples from the national survey, and 156 counties representing rural areas and districts representing urban areas in 31 provinces were selected in accordance with 10 socioeconomic, education, demographic, and health indicators. Five towns/subdistricts were randomly selected in each county/district, and then three villages/communities were randomly selected from each town/subdistrict. Finally, 60 households were randomly selected from each village/community. The response rate of the sixth NHSS was 89.2%.

**Fig. 1:**
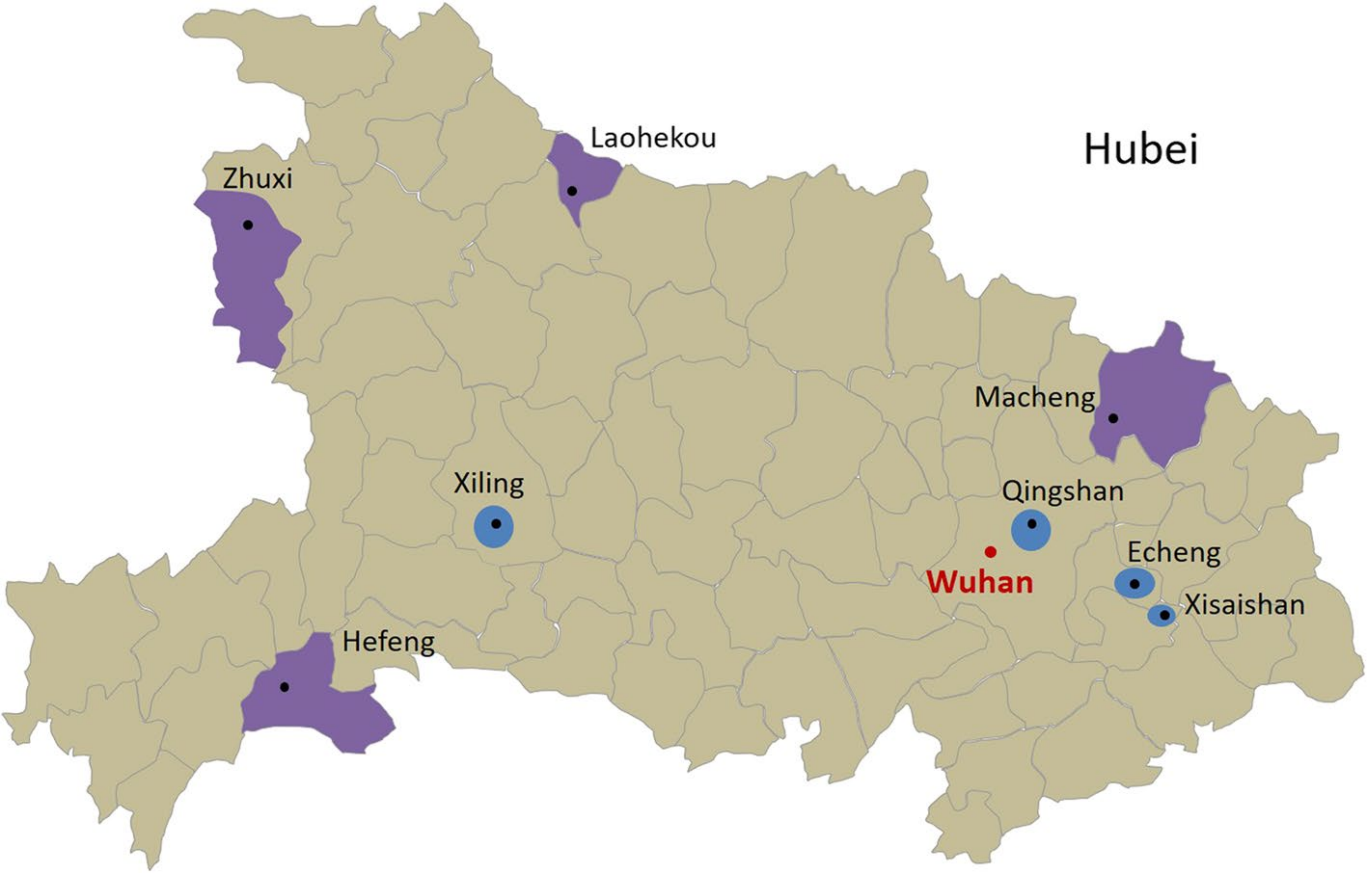
Sample areas

Hubei province is located in Central China. It has a gross regional product of USD 590 million in 2018, considered to be at the upper middle level in China. In the NHSS of Hubei Province, eight eligible regions, including four counties and four districts, were sampled. Figure 1 displays a map of Hubei Province and labels the eight sample counties and districts in purple and blue, respectively. The population, gross regional product and landform of the sample counties and districts in 2018 were presented in Additional file 1. In total, 10,987 residents participated in the survey. Among them, 1638 used inpatient services in the previous year.

### Measures

#### Bypass for inpatient care

Primary care facilities provide inpatient care in China. The government has established a diagnosis and treatment catalog for primary care facilities (i.e., township/community health center) to differentiate the scope of inpatient care between primary care facilities and hospitals. The catalog covers a number of diseases that could be diagnosed and treated at township/community health centers (Additional file 2 lists the diseases) [26, 27]. Following the measurement of bypass behavior in previous studies [28–30], bypass for inpatient care was identified in this study if the patient was discharged in a hospital (rather than a township/community health center) for a certain disease covered by the catalog mentioned above. The survey questions and categories of bypass for inpatient care were shown in Additional file 2. Among the 1638 patients who used inpatient services in the previous year, 1352 were hospitalized for diseases covered by the catalog and included in this study for further analysis.

#### Associated factors of bypass for inpatient care

Anderson’s Behavioral Model of Health Services Use (BMHSU) was adopted as a theoretical framework to analyze the determinants of bypass for inpatient care [13, 31]. According to BMHSU, utilization of health services is determined by predisposing characteristics (e.g., demographic and social factors), enabling characteristics (e.g., financing, health policy, and health system factors) and need characteristics (including perceived and evaluated health). In this study, the following factors that could be divided into three categories were included as the determinants of bypass for inpatient care: predisposing characteristics, including gender, age, marital status, and educational level; enabling characteristics, including employment status, annual family income, having a family doctor or not, and type of nearest healthcare facility; and need characteristics, including hospitalization diseases and number of chronic diseases. The definitions, survey questions and categories of the associated factors were shown in Additional file 3.

### Statistical analysis

Descriptive statistics was used to present the characteristics of samples and the rate of bypass for inpatient care. Univariate analysis was used to analyze the distribution of bypass for inpatient care on different patient characteristics. Pearson’s chi-square test was adopted for univariate analysis. Logistic regression was conducted to identify the associated factors of bypass for inpatient care among urban and rural residents. The dependent variable was whether the patient has bypass behavior or not, which was a binary variable with a value of 1 indicating bypass for inpatient care was experienced. The outliers was deleted (less than 1%) and then handled as missing data. The rate of missing value was less than 3% for all of the variables that contained missing data. We used the average value and the most common value of the responses from the other participants to fill in the missing value for continuous variables (including age and family income) and categorical variables (including level of education and type of nearest health care facility), respectively.

## Results

### Study participants

A total of 1352 patients who utilized inpatient care were included in this study. Table 1 presents the description of these participants. In total, 54% of the participants were female. The average age of all participants was 56 years old, with urban participants (58 years) were older than the rural ones (54 years). The participants with junior high school education and below accounted for 74.7%. Approximately half of the participants (44.2%) have signed a family doctor. The largest proportion of rural participants lived closest to village clinics (42.9%), while that of urban participants lived closest to hospitals (34.8%). The annual income of urban residents (USD 11025.42) was higher than that of rural residents (USD 5341.12).

**Table 1:** Description of participants

Variable	Total (%) ***n*** = 1352	Rural (%) ***n*** = 746	Urban (%) ***n*** = 606
**Sex**			
Female	732 (54.1)	408 (54.7)	324 (53.5)
Male	620 (45.9)	338 (45.3)	282 (46.5)
**Age**			
Mean (SD^a^)	55.67 (22.1)	53.61 (21.6)	58.20 (22.5)
**Education**			
JHS^b^ and below	1010 (74.7)	641 (85.9)	369 (60.9)
SHS^c^	219 (16.2)	75 (10.1)	144 (23.8)
Above college	123 (9.1)	30 (4.0)	93 (15.3)
**Marital status**			
Married	1143 (84.5)	623 (83.5)	520 (85.8)
Single	209 (15.5)	123 (16.5)	86 (14.2)
**Employment status**			
Employed	796 (58.9)	356 (47.7)	440 (72.6)
Unemployed	556 (41.1)	390 (52.3)	166 (27.4)
**Hospitalized illness**			
Respiratory diseases	292 (21.6)	177 (23.7)	115 (19.0)
Circulatory system diseases	243 (18.0)	124 (16.6)	119 (19.6)
Digestive system diseases	158 (11.7)	88 (11.8)	70 (11.6)
Skin and bone system diseases	339 (25.1)	198 (26.3)	141 (23.3)
Reproductive system diseases	199 (14.7)	113 (15.1)	86 (14.2)
Endocrine system diseases	70 (5.2)	23 (3.1)	47 (7.8)
Others	51 (3.8)	23 (3.1)	28 (4.6)
**Number of chronic diseases**	1.72 (0.924)	1.64 (0.902)	1.82 (0.946)
**Family doctors**			
Signed	597 (44.2)	366 (49.1)	231 (38.1)
Unsigned	755 (55.8)	380 (50.9)	375 (61.9)
**Type of nearest health care facility**			
Private clinic	193 (14.3)	112 (15.0)	81 (13.4)
Village clinic/community service station	467 (34.5)	320 (42.9)	147 (24.3)
Township/community health center	384 (28.4)	217 (29.1)	167 (27.6)
County hospital and above	308 (22.8)	97 (13.0)	211 (34.8)
**Annual income**			
Mean (SD)	52,160.25 (53,321.2)	35,314.40 (26,868.6)	72,897.87 (68,405.09)

^a^ *SD* Standard deviation

^b^ *JHS* Junior high school

^c^ *SHS* Senior high school

### Rate of bypass for inpatient care

Table 2 shows the rate of bypass for inpatient care in urban and rural areas. The total rate of bypass for inpatient care was 73.8%. The rate in rural areas (56.2%) was lower than that in urban areas (91.3%). In rural areas, Laohekou County showed the highest proportion of residents with bypass for inpatient care, accounting for 63.5%, while Hefeng County exhibited the lowest proportion of 41.4%. In urban areas, the highest proportion of residents with bypass for inpatient care was found in Qingshan District, accounting for 94.7%, while the lowest was identified in Xisaishan District, which accounted for 87.3%.

**Table 2:** The rate of bypass for inpatient care in sample regions in 2018

County/District	Total	Bypass for inpatient care	Bypass for inpatient care rate
Rural			
Hefeng	140	58	41.4
Laohekou	156	99	63.5
Zhuxi	170	95	55.9
Macheng	280	167	59.6
Total	746	419	56.2
Urban			
Echeng	135	120	88.9
Xiling	115	108	93.9
Qingshan	190	180	94.7
Xisaishan	166	145	87.3
Total	606	553	91.3
Total	1352	972	73.8

### Distribution of bypass for inpatient care on patient characteristics

Table 3 shows that among rural and urban patients, those who signed a family doctor showed lower rates of bypass for inpatient care than those who did not sign (56.6% vs. 67.9% for rural patients, 87.9% vs. 93.3% for urban patients). The rural and urban patients who lived closest to hospitals presented the highest rate of bypass (96.9 and 97.6%, respectively). The higher the income of patients was, the higher the rate of bypass for both patients. Rural residents with circulatory, digestive, and reproductive diseases showed the highest rates of bypass for inpatient care (67.7, 68.2, and 69.9%, respectively). Younger urban participants were more likely to experience bypass for inpatient care than the older ones. Rural patients with higher educational level showed higher rates of bypass for inpatient care. However, the distribution of bypass on educational level was not significantly different among urban patients.

**Table 3:** Distribution of bypass for inpatient care on patient characteristics

variable	Rural (n, %)		X^**2**^	***P***	urban (n, %)		X^**2**^	***P***
	Bypassers	Non-bypassers			Bypassers	Non-bypassers		
**Sex**								
Male	208 (61.5)	130 (38.5)	0.166	0.684	262 (92.9)	20 (7.1)	1.807	0.179
Female	257 (63.0)	151 (37.0)			291 (89.8)	33 (10.2)		
**Age**								
≤ 60	268 (67.0)	132 (33.0)	8.002	0.05	226 (97.8)	5 (2.2)	20.26	< 0.001
> 60	197 (56.9)	149 (43.1)			327 (87.2)	48 (12.8)		
**Education**								
JHS^a^ and below	373 (58.2)	268 (41.8)	33.863	< 0.001	333 (90.2)	36 (9.8)	4.195	0.123
SHS^b^	64 (85.3)	11 (14.7)			130 (90.3)	14 (9.7)		
Above college	28 (93.3)	2 (6.7)			90 (96.8)	3 (3.2)		
**Marital status**								
Married	387 (62.1)	236 (37.9)	0.073	0.786	468 (90.0)	52 (10.0)	7.221	0.007
Single	78 (63.4)	45 (36.6)			85 (98.8)	1 (1.2)		
**Employment status**								
Employed	214 (60.1)	142 (39.9)	1.429	0.232	404 (91.8)	36 (8.2)	0.640	0.424
Unemployed	251 (64.4)	139 (35.6)			149 (89.8)	17 (10.2)		
**Hospitalized illness**								
Respiratory diseases	93 (52.5)	84 (47.5)	18.488	0.005	102 (88.7)	13 (11.3)	11.724	0.058
Circulatory system diseases	84 (67.7)	40 (32.3)			106 (89.1)	13 (10.9)		
Digestive system diseases	60 (68.2)	28 (31.8)			66 (94.3)	4 (5.7)		
Skin and bone system diseases	118 (59.6)	80 (40.4)			123 (87.2)	18 (12.8)		
Reproductive system diseases	79 (69.9)	34 (30.1)			84 (97.7)	2 (2.3)		
Endocrine system diseases	12 (52.2)	11 (47.8)			45 (95.7)	2 (4.3)		
Others	19 (82.6)	4 (17.4)			27 (96.4)	1 (3.6)		
**Number of chronic diseases**								
None	57 (67.1)	28 (32.9)	0.924	0.630	56 (90.3)	6 (9.7)	6.734	0.034
1–2	326 (61.6)	203 (38.4)			344 (90.5)	36 (9.5)		
> 2	82 (62.1)	50 (37.9)			147 (89.6)	17 (10.4)		
**Family doctors**								
Signed	207 (56.6)	159 (43.4)	10.206	0.001	203 (87.9)	28 (12.1)	5.329	0.021
Unsigned	258 (67.9)	122 (32.1)			350 (93.3)	25 (6.7)		
**Type of nearest healthcare facility**								
Private clinic	76 (67.9)	36 (32.1)	69.105	< 0.001	77 (95.1)	4 (4.9)	25.209	< 0.001
Village/community service station	189 (59.1)	131 (40.9)			130 (88.4)	17 (11.6)		
Township/community health center	106 (48.8)	111 (51.2)			140 (83.8)	27 (16.2)		
County hospital and above	94 (96.9)	3 (3.1)			206 (97.6)	5 (2.4)		
**Annual income**								
< 50,000	356 (59.6)	241 (40.4)	10.468	0.005	223 (86.8)	34 (13.2)	12.606	0.002
50,000–80,000	61 (77.2)	18 (22.8)			119 (92.2)	10 (7.8)		
> 80,000	48 (68.6)	22 (31.4)			211 (95.9)	9 (4.1)		

^a^ *JHS* Junior high school

^b^ *SHS* Senior high school

### Determinants of bypass for inpatient care

Table 4 presents the determinants of bypass for inpatient care among rural and urban residents. Age, education level, employment status, the type of nearest health care facility and the hospitalized illness were associated with bypass bebavior in rural areas. Age and the type of nearest health care facility were associated with bypass bebavior in urban areas. The model showed that older residents were less likely to bypass than younger residents. The residents whose closest healthcare facility was hospitals were more likely to have bypass behavior in rural and urban areas than those living closest to township/community health centers. In rural areas, residents who graduated from senior high school were more likely to have bypass for inpatient care than those with lower education. Compared with residents suffering from respiratory diseases, those who suffered from circulatory, digestive, skin and bone, and reproductive diseases were likely to show bypass for inpatient care. A notable detail was that having signed a family doctor or not was not significantly associated with bypassing primary care for inpatient care at hospitals.

**Table 4:** Determinants of bypass for inpatient care among urban and rural areas

Variables	Rural		Urban	
	OR (95% CI)	***P***	OR (95% CI)	***P***
**Gender (female)**				
Male	0.839(0.590, 1.192)	0.327	0.661(0.343, 1.276)	0.218
**Age**	0.982(0.969, 0.995)	0.006	0.947(0.919, 0.976)	< 0.001
**Education (JHS and below)**				
SHS	0.213(0.046, 0.980)	0.047	0.746(0.196, 2.833)	0.666
Above College	0.698(0.134, 3.642)	0.670	0.524(0.131, 2.094)	0.361
**Married (No)**				
Yes	0.661(0.325, 1.343)	0.252	2.404(0.265,21.854)	0.436
**Employment status (Unemployed)**				
Employed	1.573(1.106, 2.239)	0.012	0.457(0.201, 1.038)	0.061
**Family income (< 50,000)**				
50,000–80,000	0.884(0.474, 1.648)	0.697	0.499(0.207, 1.204)	0.122
80,000 and above	1.740(0.767, 3.947)	0.185	0.655(0.238, 1.800)	0.412
**Family doctor (unsigned)**				
Signed	1.225(0.873, 1.718)	0.241	1.338(0.700, 2.559)	0.379
**Type of nearest health care facility (township/community health center)**^**a**^				
Private clinic	2.387(1.438, 3.962)	0.001	3.821(1.192,12.251)	0.024
Village/community service station	1.751(1.197, 2.560)	0.004	2.064(0.968, 4.403)	0.061
County hospital and above	26.091(7.867, 86.537)	< 0.001	8.323(2.936,23.591)	< 0.001
**Number of chronic diseases**	1.180(0.758, 1.837)	0.464	1.089(0.534, 2.221)	0.814
**Hospitalized illness** (**respiratory diseases)**^**a**^				
Circulatory system diseases	2.378(1.328, 4.258)	0.004	2.075(0.797, 5.402)	0.135
Digestive system diseases	2.317(1.280, 4.192)	0.006	3.661(1.025,13.080)	0.046
Skin and bone system diseases	1.758(1.088, 2.840)	0.021	1.442(0.591, 3.518)	0.421
Reproductive system diseases	1.616(0.882, 2.961)	0.121	4.967(0.956, 25.811)	0.057
Endocrine system diseases	1.104(0.420, 2.898)	0.841	3.760(0.711, 19.886)	0.119
Others	4.564(1.351, 15.413)	0.014	4.296(0.494,37.378)	0.187

## Discussion

This study showed that the rate of bypass for inpatient care (73.8%) was sizable, which is similar with that for outpatient care (85.83%), as indicated by a study among four counties in Central China [16]. In the present study, the rate of bypass for inpatient care in rural areas was 56.2%, lower than that in urban areas. However, this rate was still out of the range of 30–44% shown by previous studies on the proportion of patients who bypassed their local hospital for inpatient care services from the US [15, 32]. Such high rates of bypass for inpatient care may be due to the weakness of primary care, a lack of gate-keeping system, the increase in patients’ risk aversion, and the insufficient economic incentives from the health insurance in China. Though one important aim of the 2009 healthcare reform in China was strengthening the primary care system, a decreasing proportion of primary care providers to overall healthcare providers illustrated a relative strengthening of the hospital sector and a relative weakening of the primary care sector from 2009 to 2017 [33]. Residents generally have little trust in primary care due to both objective factors (e.g. the relatively lower staff capacity and less medical resources) and subjective factors (e.g. the fear of misdiagnosis or wrong treatment) [34, 35], and they could access any healthcare facilities without referral under China’s free-access system. To avoid misdiagnosis and delays in disease treatment, patients usually bypass primary care to seek healthcare in hospitals [34]. In addition, the compensation gap from health insurance for the same disease between primary care and hospitals is not large; thus, residents do not have sufficient financial incentives to visit primary care facilities [36]. The rate of visits to primary care facilities decreased from 62% (2009) to 54% (2017) [37], which is much lower than the value (no less than 80%) recommended by World Health Organization. Therefore, more actionable measures in strengthening primary care and increasing financial incentives to lead patients to visit primary care are needed. Gradually establishing a referral system on the basis of primary care capacity building and interacted information system between healthcare facilities is also recommended.

The rate of bypass for inpatient care among urban residents was higher than that among rural residents (91.3% vs. 56.2%). This finding was mainly due to the higher hospital accessibility in cities and the inability of some community health centers to carry out inpatient services. Urban residents are closer to higher-level health care facilities than rural residents, and the time and cost to reach higher-level health care facilities are often lower. Garnick believes that patients evaluate the time and cost of arrival when choosing a healthcare facility [38]. Thus, urban residents were more inclined to visit higher-level hospitals than rural residents. However, compared with cities, only one township health center and one village clinic, which are both primary care facilities, usually exist in each town and village, respectively. Therefore, the time and economic cost of reaching a hospital was much higher for rural residents. Although healthcare facilities with better medical quality are attractive, higher fees and longer distance limit the choice of rural residents [38, 39]. In cities, different from township health centers, which are required and able to provide inpatient care services in rural areas, only community health centers with strong health service capacities are encouraged to provide inpatient care services [26]. Therefore, the inability to provide inpatient care among community health centers was another reason for the much higher rate of bypass in urban areas.

In both areas, age and the type of nearest healthcare facility were associated with residents’ bypass behavior for inpatient care. Older residents were less likely to experience bypass, inconsistent with the result of a previous study [40]. This finding may be due to the inconvenience for the elderly to travel and be hospitalized far from home and their higher satisfaction with primary care facilities shown by previous surveys [41]. Patients who lived closest to clinics/community health stations (which are primary care facilities with smaller size and cannot provide inpatient care services) or hospitals had a higher risk of bypass for inpatient care than those who lived closest to township/community health centers. For majority of patients who lived closest to clinics, especially rural patients, township health centers may be their closest healthcare facility that is able to provide inpatient care. However, they preferred travelling out of the town for inpatient care at hospitals. This finding indicated that township/community health centers were only able to retain small areas of surrounding residents. According to Anderson’s Behavioral Model, the distribution of health care facilities and how they are structured to offer services have impacts on health service utilization of residents [42]. Therefore, it may need to be improved how different levels of health care facilities were structured to deliver services in both rural and urban areas. Surprisingly, having signed a family doctor had no significant effect on bypass for inpatient care for both patients according to logistic regression analysis. Although the family doctor contracting policy has been in place for several years, its overall effect was not satisfactory, and the restrictions on residents’ health-seeking behavior have not begun to appear because of the deep-rooted self-selection of doctors, as some research reported [43–45]. In addition, bypass for inpatient care was identified in this study if the patient was hospitalized in a hospital for a certain disease covered by a catalog (which included a list of diseases that could be diagnosed and treated at primary care facilities and was established by the government). Therefore, patients who had signed a family doctor may experience bypass for inpatient care via referral on their own request or on family doctors’ advice as a result of relatively lower staff capacity and lower quality of inpatient care at primary care facilities [34, 35]. Among rural patients, those with circulatory, digestive, or skin and bone system diseases were more likely to show bypass behavior than those with respiratory diseases, possibly indicating that the inpatient care services for circulatory, digestive or skin and bone system diseases at primary care facilities could not meet the health care needs of patients and requires further improvement. However, the result must be interpreted with caution because other factors (e.g. severity of illness or patient satisfaction) may also lead to the higher possibility of bypass behavior among patients with circulatory, digestive or skin and bone system diseases. This needs to be further studied.

## Conclusion

In this study, the rate of bypass for inpatient care was estimated, and the determinants of bypass for rural and urban residents were explored. The results showed that bypass for inpatient care was sizable, and urban residents had a higher bypass rate for inpatient care than rural residents. Age and the type of nearest healthcare facilities were associated with bypass for inpatient care. However, whether having signed a family doctor or not had no significant effect on the bypass behavior for both types of patients. Disease was related to bypass only in rural areas. More actionable measures in strengthening and leading patients to primary care are needed. Gradual establishment of a referral system is also recommended. Inpatient care services for circulatory, digestive, or skin and bone system diseases may be prioritized for improvement at primary care facilities to reduce bypass behavior in rural China.

## Limitations

Though some community health centers in urban areas were not able nor required by the government to provide inpatient care services (which may result in the high rate of bypass) before 2018, the analysis for urban residents was still included in this study. The reason is because community health centers were encouraged to provide inpatient care by the government by publishing the standards for capacity building in delivering services for primary care facilities through a campaign called Delivering Qualified Services at Primary Care Facilities, in 2018. Therefore, understanding the bypass rate for inpatient care and the contributing factors that may guide the implementation of the campaign is still worthwhile. In addition, a few potential contributing factors (e.g., patient satisfaction, severity and duration of illness, quality of care) were not included due to lack of data. Some contextual characteristics included in Anderson’s Behavioral Model (e.g. community values and cultural norms regarding where to obtain health care, financial incentives from health insurance to reduce bypass behavior) may also have impact on bypassing, which were not addressed in this study. Further studies on the other contributing factors of bypass for inpatient care in China are needed.

## Supplementary Information

**Additional file 1.** The population, gross regional product and landform of the sample counties and districts in 2018.

**Additional file 2.** Service capacity standard of Township/Community Health Service Center(2018 Edition).

**Additional file 3.** Definitions, survey questions and categories of bypass for inpatient care and associated factors.

## Data Availability

The datasets used and analyzed in this study are available from the corresponding author upon reasonable request.

## Abbreviations

NHSS: National Health Service Survey
BMHSU: Anderson’s Behavioral Model of Health Services Use.
