# Supplementary material for: Difference in bypass for inpatient care and its determinants between rural and urban residents in China
Source: Int J Equity Health. 2022 Sep 13;21:132. doi: 10.1186/s12939-022-01734-0 (PMC9469557; doi:10.1186/s12939-022-01734-0)
Supplement: Supplementary file 1 — Additional file 1. The population, gross regional product and landform of the sample counties and districts in 2018. [file 12939_2022_1734_MOESM1_ESM.docx]

**Additional file 1**

**Table S1 The population, gross regional product and landform of the sample counties and districts in 2018**

| **County/District** | | **Population (Thousands)** | **Gross regional product (USD: million)** | **Gross regional product per capita (USD)** | **Landform** |
| --- | --- | --- | --- | --- | --- |
| Rural | Hefeng | 205 | 927.10 | 4485.37 | Mountainous |
|  | Laohekou | 482 | 5518.75 | 11367.60 | Plain and hilly |
|  | Zhuxi | 315 | 1244.71 | 3917.80 | Hilly and mountainous |
|  | Macheng | 880 | 4615.84 | 5202.27 | Hilly and mountainous |
| Urban | Echeng | 314 | 3951.90 | 12495.86 | Plain, hilly and mountainous |
|  | Xiling | 405 | 5657.89 | 13855.56 | Plain and hilly |
|  | Qingshan | 575 | 9479.72 | 16345.88 | Plain |
|  | Xisaishan | 233 | 2983.97 | 12702.36 | Plain and hilly |
