# Supplementary material for: Difference in bypass for inpatient care and its determinants between rural and urban residents in China
Source: Int J Equity Health. 2022 Sep 13;21:132. doi: 10.1186/s12939-022-01734-0 (PMC9469557; doi:10.1186/s12939-022-01734-0)
Supplement: Supplementary file 2 — Additional file 2. Service capacity standard of Township/Community Health Service Center(2018 Edition). [file 12939_2022_1734_MOESM2_ESM.docx]

**Additional file 2**

**Table S2 Service capacity standard of Township/Community Health Service Center**

**(2018 Edition)**

| **Profession** | **Disease** | **ICD code** |
| --- | --- | --- |
| Internal Medicine department | hypertension | I10.x00 |
|  | Coronary atherosclerotic heart disease | I25.103 |
|  | Vertigo syndrome | H81.901 |
|  | Migraine | G43.900 |
|  | Emphysema | Q24.900 |
|  | Myocarditis | I51.400 |
|  | Stroke | I64.x00 |
|  | Acute bronchitis | J04.100 |
|  | bronchitis | J40.x00 |
|  | pneumonia | J18.900 |
|  | Chronic cor pulmonale | I27.900 |
|  | Acute upper respiratory tract infection | J06.900 |
|  | diarrhea | K52.916 |
|  | Gastroenteritis | A09.901 |
|  | colitis | A09.902 |
|  | cholecystitis | K81.900 |
|  | Acute glomerulonephritis | N00.902 |
|  | anemia | D64.900 |
|  | Urinary tract infection | N39.000 |
|  | diabetes | E14.900 |
|  | Hyperlipidemia | E78.500 |
|  | Transient ischemic attack | G45.900 |
|  | Shingles | B02.900 |
|  | dermatitis | L30.900 |
|  | tuberculosis | A16.200 |
| surgical department | appendicitis | K37.x00 |
|  | Inguinal hernia | K40.900 |
|  | Hydrocele | N43.301 |
|  | hemorrhoid | I84.900 |
|  | Perianal abscess | K61.001 |
|  | Benigprostatic hyperplasia | N40.x00 |
|  | Head trauma | S09.900 |
|  | fracture | T14.200 |
|  | Frozen shoulder | M75.001 |
|  | arthritis | M13.900 |
|  | Lumbar muscle strain | M54.505 |
|  | Lumbar disc herniation | M51.202 |
|  | stomach ache | R10.400 |
|  | Bile duct stones | K80.500 |
|  | Urinary stones | N20.900 |
|  | constipate | K59.000 |
|  | Vertebral artery type cervical spondylosis | M47.001+ |
| Obstetrics and Gynecology Department | Female pelvic inflammatory disease | N73.902 |
|  | Cervical Inflammatory Disease | N72.x00 |
|  | Acute vaginitis | N76.000 |
|  | Endometritis | N71.902 |
|  | Salpingitis | N70.904 |
|  | childbirth | O83.900 |
|  | Ovarian | N70.903 |
| Eye, Otorhinolaryngology | Conjunctivitis | H10.900 |
|  | Acute nasopharyngitis | J00.x00 |
|  | Acute sinusitis | J01.900 |
|  | Nose bleeding | R04.000 |
|  | Acute tonsillitis | J03.900 |
|  | Acute pharyngitis | J06.000 |
|  | Acute pharyngitis | J02.900 |
|  | Herpetic angina | B08.501 |
|  | Otitis media | H66.900 |
|  | Non-suppurative otitis media | H65.900 |
|  | Caries | K02.900 |
| department of stomatology | Acute periodontitis | K05.200 |
|  | Partial dentition missing | K08.104 |
|  | Stomatitis | K12.112 |
|  | Purulent gingivitis | K05.101 |
|  | Oral mucosal ulcer | K12.109 |
