# Supplementary material for: Difference in bypass for inpatient care and its determinants between rural and urban residents in China
Source: Int J Equity Health. 2022 Sep 13;21:132. doi: 10.1186/s12939-022-01734-0 (PMC9469557; doi:10.1186/s12939-022-01734-0)
Supplement: Supplementary file 3 — Additional file 3. Definitions, survey questions and categories of bypass for inpatient care and associated factors. [file 12939_2022_1734_MOESM3_ESM.docx]

**Additional file 3**

**Table S3 Definitions, survey questions and categories of bypass for inpatient care and associated factors**

| **Factors** | **Definitions, survey questions and categories** |
| --- | --- |
| Bypass for inpatient care | Bypass for inpatient care was identified if the patient was discharged in a hospital (rather than a township/community health center) for a certain disease covered by the catalog (which covers a number of diseases that could be diagnosed and treated at township/community health centers and was established by the government).  Q ^a^: (1) What’s the discharge diagnosis of the admission in the previous year (if the patient was hospitalized)?  (2) Which type of health care facility were you admitted? ^b^  C ^c^: 0=Without bypass, 1=with bypass |
| ***Predisposing characteristics*** | |
| Age | Age of the patient (continuous variable)  Q ^b^: What’s your date of birth? |
| Gender | Gender of the patient  Q: What’s your gender?  C: 0=Female, 1=Male |
| Education | Education level of the patient  Q: What’s your level of education?  C: 0=Junior high school and below, 1=Senior high school, 2=Above College |
| Marital status | Marital status of the patient  Q: What’s your marital status?  C: 0=Single/divorced/widowed, 1=Married |
| ***Enabling characteristics*** | |
| Employment status | Employment status of the patients  Q: What’s your employment status?  C: 0=Unemployed/retired/student, 1=Employed |
| Family doctor | Whether the patient have signed a family doctor or not  Q: Have you signed a family doctor?  C: 0=Unsigned, 1=Signed |
| Family income | Family income of the patient (continuous variable)  Q: What’s the total income of your family in the previous year? |
| Type of nearest health care facility | The nearest health care facility to the patient’s home  Q: What’s the nearest health care facility to your home?  C: 0=Township/community health center,  1=Private clinic,  2=Village/community service station,  3=County hospital and above |
| ***Needs characteristics*** | |
| Number of chronic diseases | Number of chronic diseases of the patient (count variable)  Q: (1) Are you diagnosed with hypertension?  (2) Are you diagnosed with diabetes?  (3) Are you diagnosed with other chronic diseases? What are they? ^d^ |
| Hospitalized illness | The hospitalized illness of the patients in the previous year  Q: What’s the discharge diagnosis of the admission in the previous year (if the patient was hospitalized)?  C: 0=Respiratory diseases, 1=Circulatory system diseases, 2=Digestive system diseases, 3=Skin and bone system diseases, 4=Reproductive system diseases, 5=Endocrine system diseases, 6=Others |

^a^ Q: the question from the sixth National Health Service Survey (NHSS) of Hubei Province in 2018.

^b^ Bypass for inpatient care was identified using two questions from the NHSS.

^c^ Categories: the categories used in the regression model.

^d^ The number of chronic disease was identified using three questions from the NHSS.
